# Stop

**Published:** 1877-12

**Authors:** 


### STOP!

We have recently had the pleasure of examining an ingenious contrivance invented by Dr. E. Osmond of this city, which places under control of the operator the motion of the burring engine. It consists of a division of that portion of the shaft which runs through the hand piece. The end of the main shaft is grooved for the reception of a tongue upon the connecting end of the part carrying the bur. These two parts are held in apposition by means of a spiral spring, and are thrown out of gear by means of a little nut under the control of the fore finger which relieves the bearing of the spring and stops the motion instantaneously. This stop motion is a very useful one as it does away with the many annoyances which aggravate the operator, such as the bur catching the rubber dam, winding it around the shaft and tearing it. It further admits of the bur being used as a probe thereby saving time, and the inconvenience of changing instruments. The Dr. has several other valuable improvements in course of perfection which we hope will be placed before the profession with as much energy as he displays in working them up.

